# Eukaryotic rRNA Modification by Yeast 5-Methylcytosine-Methyltransferases and Human Proliferation-Associated Antigen p120

**DOI:** 10.1371/journal.pone.0133321

**Published:** 2015-07-21

**Authors:** Gabrielle Bourgeois, Michel Ney, Imre Gaspar, Christelle Aigueperse, Matthias Schaefer, Stefanie Kellner, Mark Helm, Yuri Motorin

**Affiliations:** 1 Laboratoire IMoPA, UMR 7365 UL-CNRS, BioPole de UL, Vandoeuvre-les-Nancy, France; 2 EMBL Heidelberg, Meyerhofstraße 1, 69117, Heidelberg, Germany; 3 Division of Epigenetics, German Cancer Research Center (DKFZ), Heidelberg, Germany; 4 Institute of Pharmacy and Biochemistry, Johannes Gutenberg-University Mainz, Mainz, Germany; The John Curtin School of Medical Research, AUSTRALIA

## Abstract

Modified nucleotide 5-methylcytosine (m^5^C) is frequently present in various eukaryotic RNAs, including tRNAs, rRNAs and in other non-coding RNAs, as well as in mRNAs. RNA:m^5^C-methyltranferases (MTases) Nop2 from *S*. *cerevisiae* and human proliferation-associated nucleolar antigen p120 are both members of a protein family called Nop2/NSUN/NOL1. Protein p120 is well-known as a tumor marker which is over-expressed in various cancer tissues. Using a combination of RNA bisulfite sequencing and HPLC-MS/MS analysis, we demonstrated here that p120 displays an RNA:m^5^C- MTase activity, which restores m^5^C formation at position 2870 in domain V of 25S rRNA in a *nop2Δ* yeast strain. We also confirm that yeast proteins Nop2p and Rcm1p catalyze the formation of m^5^C in domains V and IV, respectively. In addition, we do not find any evidence of m^5^C residues in yeast 18S rRNA. We also performed functional complementation of Nop2-deficient yeasts by human p120 and studied the importance of different sequence and structural domains of Nop2 and p120 for yeast growth and m^5^C-MTase activity. Chimeric protein formed by Nop2 and p120 fragments revealed the importance of Nop2 N-terminal domain for correct protein localization and its cellular function. We also validated that the presence of Nop2, rather than the m^5^C modification in rRNA itself, is required for pre-rRNA processing. Our results corroborate that Nop2 belongs to the large family of pre-ribosomal proteins and possesses two related functions in pre-rRNA processing: as an essential factor for cleavages and m^5^C:RNA:modification. These results support the notion of quality control during ribosome synthesis by such modification enzymes.

## Introduction

Post-transcriptional RNA modification is an integral part of global RNA maturation in all cell types. During this step, single enzymes or enzymatic systems form numerous chemically distinct modified residues. The great majority of RNA modifications are methylations, consisting of the transfer of a methyl group from S-adenosyl-L-methionine (SAM) to various positions in the base or 2'-OH of ribose [1].

Among these modifications, methylation of cytosine at position 5 is one of the most widespread. Indeed, m^5^C was first found in DNA, where it plays an essential role in epigenetic regulation [2] and also in different RNAs. In Bacteria, m^5^C was found in both 16S and 23S rRNA [3], while in Eukarya, this modified residue was first reported in Large SubUnit (LSU) rRNA and tRNAs [4–6]. Two sites of m^5^C modification in human and *Xenopus laevis* 28S rRNA were mapped by classical RNA sequencing techniques [6] and two corresponding sites were recently mapped in domains IV and V of yeast 25S rRNA [4, 5, 7, 8], for review [9]. Bisulfite-mapping of m^5^C residues in human RNAs at whole genome scale was also reported [10]. Over 10,000 potential sites of m^5^C modification were mapped in whole human transcriptome, a great part of located residues was found in mRNA, non-coding RNAs and tRNAs. Two sites of m^5^C modification were confirmed for human 28S rRNA, even if additional clusters of non-deaminated C were detected. Presently, there is no evidence for the presence of m^5^C modifications in human or yeast 5S and 18S rRNA [11].

In all living organisms, methylation of C to m^5^C is catalyzed by specific enzymes, which belong to the so-called Fmu family in Bacteria and to NSUN proteins in higher eukaryotes [9]. In yeast, three enzymes that methylate cytosine have been reported: Trm4 (Ncl1) was previously characterized as tRNA-specific MTase, acting at positions 34/40/48/49 in different tRNAs [12, 13]. Recently, Nop2 and Rcm1 have been reported to catalyze m^5^C formation in LSU yeast rRNA (see Table 1) [7, 8].

**Table 1:** Known and putative RNA:m^5^C-MTases in *E*. *coli*, *S*. *cerevisiae* and *H*. *sapiens*.

*E*. *coli*	*S*. *cerevisiae*	*H*. *sapiens*
**RsmB (Fmu)** 16S rRNA, m^5^C_967_		
**RsmF (YebU)** 16S rRNA, m^5^C_1407_		
**RlmI (YccW)** 23S rRNA, m^5^C_1962_		
	**Nop2** 25S rRNA, m^5^C_2870_	**p120 (NSUN1/Nol1)** 28S rRNA, m^5^C_4447_
	**Trm4 (Ncl1)** tRNA, m^5^C_34/40/48/49_	**hTrm4 (Misu/NSUN2)** tRNA, m^5^C_34/48/49/50_
		**Dnmt2** tRNA, m^5^C_38_
	**Rcm1** 25S rRNA, m^5^C_2278_	**NSUN5A, B and C** 28S rRNA, m^5^C_3761_
		**NSUN3**
		**NSUN4** mito 12S rRNA, m^5^C_911_
		**NSUN6**
		**NSUN7**

Putative positions of m^5^C are underlined.

The human genome contains at least 9 genes encoding putative RNA:m^5^C-MTases which share sequence similarities with yeast Nop2/Trm4/Rcm1 (so called NSUN/NOP2/NOL1 family). These proteins play important roles in multiple cellular functions. Proteins p120 (NSUN1/NOL1), NSUN2 and NSUN4 participate in cell proliferation and differentiation and may play an important role in cancer [14–19]. In addition, NSUN2 dysfunction may induce intellectual disability [20]. NSUN7 probably cause male sterility [21]. Physiological functions of NSUN3, NSUN5 and NSUN6 are still unknown. Despite the conservation and involvement of these proteins in basic mechanisms, very few investigations were conducted on these enzymes.

In humans, only the Trm4 homolog (hTrm4/Misu/NSUN2) was extensively characterized and shown to catalyze m^5^C 34/48/49/50 formation in human tRNAs [12, 17, 22, 23]. Recently, NSUN4 was characterized as a dual function protein involved in mitochondrial 12S rRNA methylation at position 911 and, in the complex with MTERF4 during ribosomal assembly [24].

The human proliferation-associated nucleolar antigen p120 exhibits important similarity to yeast Nop2 [25]. Protein p120 was discovered through a systematic analysis of antibodies directed against proteins present in the nucleoli of cancer cells and tumor tissues [26]. Whereas protein p120 is almost undetectable in normal tissues [26, 27], it is over-expressed in virtually all types of cancer cells and is therefore considered to be a predictive cancer marker [16, 28, 29]. Its concentration varies during the cell cycle, reaching its maximum value in the G2 phase [28, 30], and it was also found to be required for the G1/S transition [31]. Mouse fibroblasts transfected with a construct expressing p120 grow rapidly, and promote tumor formation when injected into mice [32, 33]. Protein p120 is localized to nucleoli and nuclear as well as nucleolar localization signals (NLS and NoLS, respectively) were found to be located in its N-terminal domain [34]. The N-terminal domain of p120 is also rich in arginine and has a high-affinity for rRNA [35].

In addition to their RNA modification enzymatic activities, many RNA:modification enzymes have additional cellular functions. For instance Dim1 [36], Spb1 [37], Bud23 [38, 39], Nep1 [40–42] and Nop2 [43] were demonstrated to be involved in pre-rRNA processing. The yeast *S*. *cerevisiae* nucleolar protein Nop2 was shown to be required for production of 25S rRNA and, consequently, during the biogenesis of 60S ribosomal subunits [43, 44]. The specificity of action of these enzymes in the pre-rRNA processing pathway is still poorly understood.

In this study, we used a combination of LC-MS/MS and RNA bisulfite sequencing to analyze the presence of m^5^C residues in yeast 18S and 25S rRNAs to confirm that Rcm1 and Nop2 catalyze m^5^C formation in 25S rRNA domains IV and V, respectively. When expressed in a Nop2-deficient yeast strain, human proliferation-associated antigen p120 as well as hybrid proteins composed of the Nop2 N-terminal domain and p120 MTase domain restored m^5^C formation in domain V of endogenous yeast 25S rRNA. These results clearly demonstrate that human proliferation associated antigen p120 is implicated in maturation and modification of eukaryotic LSU rRNA.

## Materials and Methods

### Yeast strains and media

The diploid *S*. *cerevisiae* BY4743 strain carrying a disruption of the *NOP2* gene (*nop2Δ*::*Kan* ^*r*^, denoted *nop2Δ*) was obtained from the Biovalley collection (YSC1021-673611). The haploid *rcm1Δ* strain is viable and was purchased from EUROSCARF collection (Y05348). Standard *S*. *cerevisiae* growth and handling techniques were employed. Media used were yeast extract/peptone supplemented either with 2% glucose (YPD) or galactose (YPG), and minimal medium (0.67% *Yeast Nitrogen Base*, appropriate amino acids, 2% galactose), containing 5 mg/L or 200 mg/L of Geneticin (G_418_). Transformation was carried out using the LiOAc/PEG method [45]. For complementation assays, *nop2Δ* strain was transformed with p416GalS constructs and induced to sporulate. Tetrads were dissected on YPG and at least 3 complete tetrads were analyzed. Spore colonies that were both Ura^+^ and G_418_ resistant were selected for further experiments.

### Plasmids for the complementation assays

All Nop2/p120 and hybrid protein variants were cloned into the p416GalS yeast expression plasmid (URA3 auxotrophic marker) under the control of inducible GalS promoter.

The *S*. *cerevisiae NOP2* gene was amplified by PCR using genomic DNA from the BY4742 strain. 5'- and 3'-oligonucleotides used for amplification introduced *Nhe*I and *Bam*HI restriction sites, respectively. The fragment was cloned into pET-28b (Novagen) and further subcloned into p416GalS (*Xba*I-*Nhe*I/*Xho*I). *NOL1* (*Homo sapiens* nucleolar protein 1, RZPD clone ID: IRAUp969G1110D) was obtained from RZPD (Deutsches Ressourcenzentrum für Genomforschung GmbH, Germany). The p120 sequence was amplified by PCR using pOBT7-NOL1, cloned into *Sma*I-digested pUC18 and then sub-cloned into p416GalS (*Sma*I/*Sal*I). Other protein constructs were obtained by PCR amplification using p416/NOP2 and p416/p120 templates, followed by cloning into p416GalS digested with *Sma*I.

### Subcellular localization of Nop2, p120, Nop2Δ(1–220) and HYB proteins

Sequences of the *S*. *cerevisiae NOP2* gene and of HYB were amplified by PCR using p416GalS-NOP2 and p416GalS-HYB constructs as templates. 5'- and 3'-oligonucleotides used for amplification introduced SalI restriction sites. The resulting fragments were cloned into pPS808 downstream GFP-encoding sequence. Sequences of p120 and Nop2Δ(1–220) were directly subcloned from p416GalS constructs into pPS808 by *Xba*I, *Bam*HI and *Spe*I/*Sal*I digestion respectively.

Yeast expressing a GFP fusion of truncated Nop2 fragments or Nop2-p120 hybrid were fixed in 70% ethanol for one minute and stained with 4', 6-diamidino-2-phenylindole (DAPI 2 μg/ml) for five minutes and were subsequently mounted in 4% low melting point agarose on a slide to restrict thermal motion and drifting of the cells. To exclude fixation artifacts, another batch of cells was imaged *in vivo*. In order to facilitate DNA staining in living yeast, cells were grown for 2 h in YPG+adenosine media in the presence of 20 μg/ml DAPI. However *in vivo* DAPI incorporation into the nuclei–unlike into mitochondria—was not sufficient to reliably identify the nucleus. Cells were harvested, resuspended in phosphate buffered saline and mounted on slides as described above. Imaging was carried out on a Zeiss LSM 710/780 confocal microscope, followed by quantification using ImageJ software. Nucleolar co-localization of proteins was verified using a haploid yeast strain containing a chromosomally integrated copy of Nop56 (Sik1)-mRFP [46].

### In vitro transcription

RNAs were generated by run-off transcription with T7 or SP6 RNA polymerase from the appropriate DNA template. The pTFM plasmid carrying the synthetic gene of yeast tRNA^His^ (GUG) linearized by BstNI was used as template for a fragment of tRNA^His^. rRNA fragment was prepared by PCR amplification using yeast genomic DNA, digest with NotI and fill-in by Klenow fragment (Fermentas, France), followed by cloning downstream of the T7 promoter into pBluescript II KS+. pBluescript II KS+ was linearized by EcoRI for transcription. Oligonucleotides used for DNA amplification to transcribe a fragment of domain V of 25S rRNA were 5’-CAGTGGGAATCTCGTTA-3’ and 5’-GAAAGTGATGTTGACGCAATGTGA-3’. Oligonucleotides used to amplify DNA to transcribe a fragment of domain IV of 25S rRNA were 5’- AATTCTGCTTCGGTATGAT-3’ and 5’-GTTACCACAGGGATAAC-3’. Template SLS2/E RNA was obtained by amplification using L3-U1 construction [47] with following oligonucleotides 5’-CGCGATTTAGGTGACACTATAGAAGATCTGCTGTTTAT-3’ (carrying SP6 promoter) and 5’-GTTGCTCTCCTCTGTTG-3’.

### Protein expression and purification

Recombinant protein expression of Nop2 and the N-terminal domain (1–220) alone was performed using pET28b derived plasmids transformed into *E*. *coli* BL21 (DE3) CodonPlus cells. Expression was performed at 20°C in the auto-inducible medium ZYP5052 prepared as described [48].

For preparation of the full-length Nop2 protein, cells were lysed in buffer 1 (20 mM MOPS, 50 mM NaCl, 1 mM β-mercaptoethanol, complete protease inhibitors cocktail (Roche Diagnostic, France) at pH 6.8). The S30 supernatant was obtained by centrifugation at 30,000g at 4°C. Nucleic acids present in S30 were precipitated by 0.0125% of polyethyleneimine under rotation for 5 minutes at 4°C followed by centrifugation at 30,000g to remove insoluble material. The supernatant was applied to a Q-Sepharose High Performance followed by affinity chromatography on Ni-NTA-Sepharose. Elution fractions were dialyzed against storage buffer (20 mM Tris-HCl, 100 mM NaCl, 50% glycerol at pH 7.5). Nop2 was conserved at -20°C with 1 mM DTT.

To prepare the N-terminal domain of Nop2 (1–220), cells were lysed in buffer 2 (20 mM Tris-HCl, 1 M NaCl, 20 mM imidazole at pH 7.5). The S30 supernatant was prepared as indicated above followed by precipitation of nucleic acids by 0, 0125% of polyethyleneimine for 5 min at 4°C. Insoluble material was removed by centrifugation at 30,000g. Proteins were purified by affinity chromatography on Ni-NTA Sepharose and then dialyzed against storage buffer (see above) and conserved at -20°C with 1 mM DTT.

### Ni-NTA Sepharose pull-down

RNA transcripts were 5’-end labeled with [γ-32P]ATP (3000 Ci/mmol) and purified on denaturing 8% polyacrylamide gels. Transcripts were incubated with 3 pmol of Nop2 or the N-terminal domain of Nop2 (1–220) in shift buffer (0.5 μg/μL tRNA, 10 mM Tris-HCl, 100 mM NaCl, 2.5 mM MgCl2 at pH 7.4) in 20 μL. The RNA/ protein complex was formed at 30°C for 10 minutes then incubated on Ni-NTA Sepharose beads washed with pull-down buffer (10 mM Tris-HCl, 150 mM NaCl, 0.1% Igepal at pH 8) at room temperature for 10 minutes. Beads were washed three times for 5 min at room temperature with 1 mL of pull-down buffer supplemented with 1 mM DTT and 20 mM imidazole. Supernatant was discarded and beads were resuspended by vortexing in 5 μL of SBL (62.5 mM Tris-HCl, 2% SDS, 10% glycerol, 100 mM β-mercaptoethanol, 0.01% Bromophenol blue at pH 6.8). Samples were fractionated on 8% SDS-PAGE gels and analyzed by autoradiography. Experiment was repeated three times using different batches of transcripts and purified recombinant proteins.

### Bisulfite rRNA sequencing

RNA bisulfite sequencing was performed as described in [49]. In brief, purified rRNA were treated with EpiTect Bisulfite Kit (Qiagen) in 5 cycles, precipitated and used for cDNA synthesis with deamination-specific rRNA oligos. Barcoded adapters compatible with Roche 454 GX system were added in the second PCR amplification and purified amplicons were subjected to sequencing. Sequencing was performed at least two or more times, especially when low read number was obtained.

### HPLC-MS/MS analysis

Quantification of m^5^C residues in ribosomal RNA by LC-UV-MS/MS was performed as described in [50]. In brief, gel-purified RNA was digested by nuclease P1 and alkaline phosphatase and the resulting nucleosides separated by C18 reverse phase chromatography. Optical density of the column effluent was first measured at 254 nm and redirected to electrospray ion source. MS was operated in positive mode to monitor the specific mass transition for m^5^C. MS peaks were integrated and corrected by calculated response factor from calibration curves without the use of internal standard. Analysis was performed twice with independent preparations of yeast rRNA.

## Results

### Human p120 partially rescues growth of nop2Δ yeast strain

**Fig 1:**
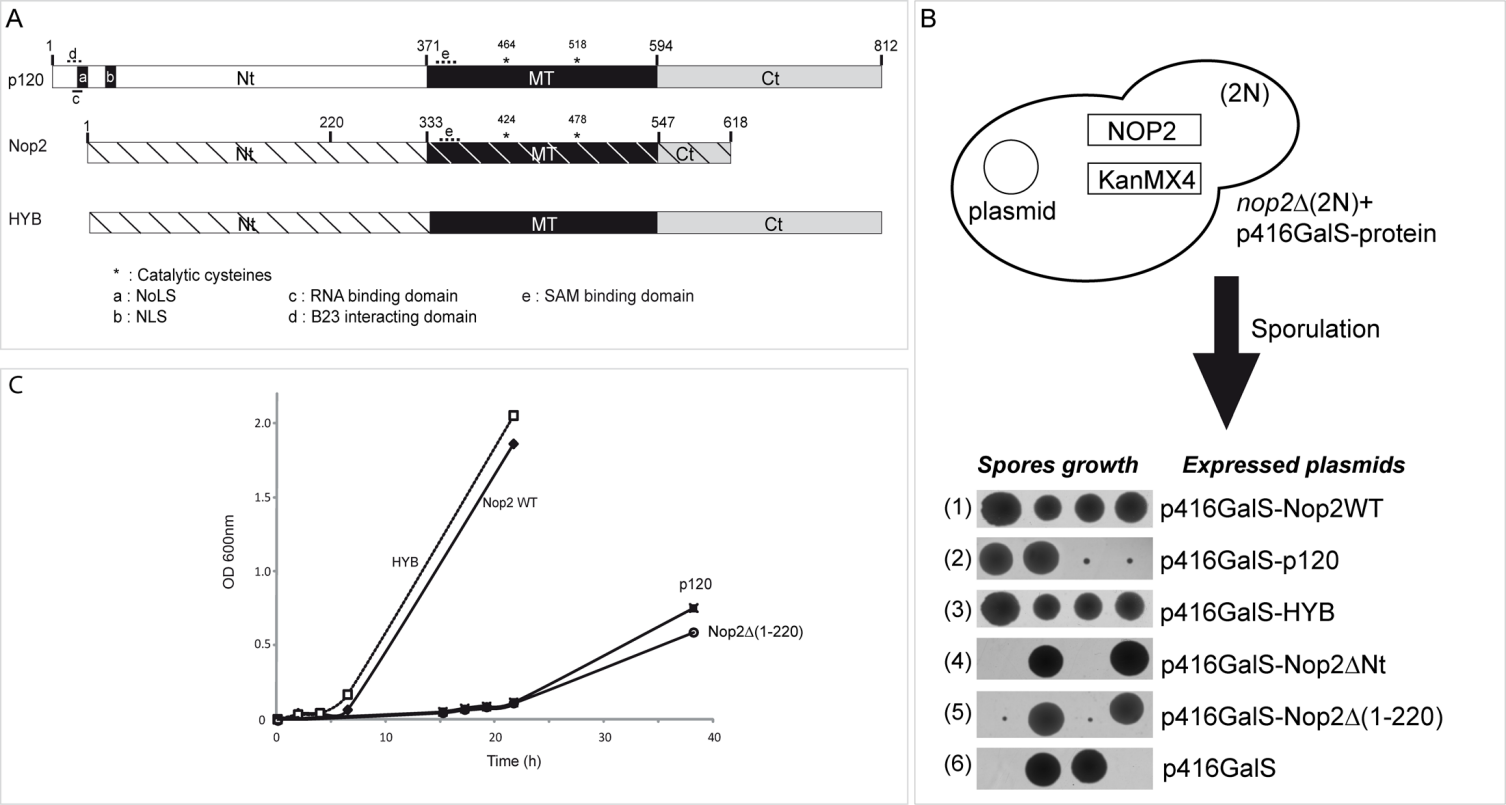
Protein domain structures of human p120 and *S*. *cerevisiae* Nop2 and conservation of the putative RNA:m^5^C-MTase active site. (A) Global alignment of human p120, yeast Nop2 and HYB protein. Proteins are represented to scale and domains present in p120 and Nop2 as well as their positions are indicated (NTD: N-terminal domain, MTD: RNA:MTase catalytic domain, CTD: C-terminal domain). The positions of catalytic cysteine residues in the MTD are indicated by asterisks. Specific sequence motifs, identified in p120 and Nop2, are indicated by letters: a: p120 Nucleolar Localization Signal (NoLS), b: p120 Nuclear Localization Signal (NLS), c: RNA-binding domain, d: B23-interacting domain, e: AdoMet (SAM) binding domain. In HYB protein, p120 structural domains are indicated as grey and black boxes and the corresponding Nop2 domains are indicated as hatched white rectangle. (B) Preparation of haploid Nop2Δ strains by sporulation and tetrad dissection. Growth of individual spores expressing different variants is shown at the bottom. (C) Growth of viable complemented haploid strains in liquid YPG medium. Strains were inoculated at 0.05−0.1 units OD600 and grown for a maximum of 40 hours on a shaker at 30°C.

The human proliferation-associated nucleolar antigen p120 belongs to a large family of putative RNA:m^5^C-MTases and its MTase domain displays approximately 60% sequence identity with Nop2 at the amino acid sequence level. Based on sequence alignment of numerous Nop2 homologs and also on the crystal structure of the RNA:m^5^C-MTase catalytic domain of human protein NSUN5 (PDB accession number 2B9E), we identified three well-defined protein domains in Nop2 and p120 (Fig 1A). In this study, they are referred to as the NTD (N-terminal domain), MTD (RNA:MTase catalytic domain) and CTD (C-terminal domain). The RNA:m^5^C-MTase catalytic domains in Nop2 and p120 encompass residues 333−547 and 371−594, respectively, and contain the universally conserved Pro-Cys-Ser (PCS) and Thr-Cys-Ser (TCS) sequences (S1A Fig) [51]. Until now, no experimental evidence was provided as to whether human p120 is a true functional homolog of yeast Nop2. Therefore we have tested the ability of human p120 to complement for Nop2 function by expressing human p120 gene under the control of the GAL promoter in Nop2-deficient *S*. *cerevisiae*. Since the deletion of *NOP2* is lethal (Fig 1B, tetrad 6), a diploid yeast strain bearing a monoallelic *NOP2* → Kan^r^ replacement was transformed with plasmids expressing Nop2, human p120, or their variants, and induced to sporulation. The resulting tetrads were dissected and spores were grown on YPG agar media to allow the expression of Nop2 or p120 proteins, under the control of the GalS promoter. Expression of the WT Nop2 gene rescued normal growth in yeast (Fig 1B, tetrad 1). Expression of human p120 protein partially complemented the growth of the yeast *nop2*Δ mutant. Despite a strong growth phenotype, viable slow-growing colonies could be obtained. The effects on cell growth were further confirmed by measuring the growth in YPG liquid medium (Fig 1C). *Nop2*-deficient yeasts expressing the p120 gene exhibited a significant lag-phase and, in addition, an increased doubling time. The slow growth phenotype observed for haploid yeast strains expressing human p120 was not related to the potential toxicity of the heterologous protein, as normal growth was restored upon co-expression of WT Nop2 (data not shown). These results suggest that the human p120 is a functional homologue of yeast Nop2.

### NTD of Nop2 is essential for efficient complementation by p120 in yeast

Analysis of p120 and Nop2 sequences revealed that NTD and CTD domains are much less conserved than MTD, which may reflect additional non-conserved cellular functions of both proteins in yeast and in human cells. Interestingly, when an NTD-truncated Nop2 variant was expressed in *nop2Δ* diploid strain, a 2:2 spore distribution was obtained after sporulation, demonstrating the lethality of this deletion (Fig 1B, tetrad 4). Moreover, expression of NTD alone in *nop2Δ* did not restore growth (data not shown). Differences in amino acid sequence between NTDs of Nop2 and p120 could explain the growth defect of the *nop2Δ* expressing p120 strain. To understand the function of these different protein domains, we designed Nop2-p120 chimera protein. The NTD of Nop2 was linked to MTD and CTD of p120 to generate a hybrid protein (HYB) (Fig 1A). This chimeric variant complemented *nop2*Δ yeast cells to the same extent as the WT Nop2 and thus more efficiently than human p120 itself (Fig 1). Interestingly, when the CTD domain of p120 was removed, this did not influence the complementation efficiency (data not shown). Altogether, this supports the notion that the NTD domain from Nop2, as long as a MT domain follows it, is essential for its efficient functional complementation in yeast.

**Fig 2:**
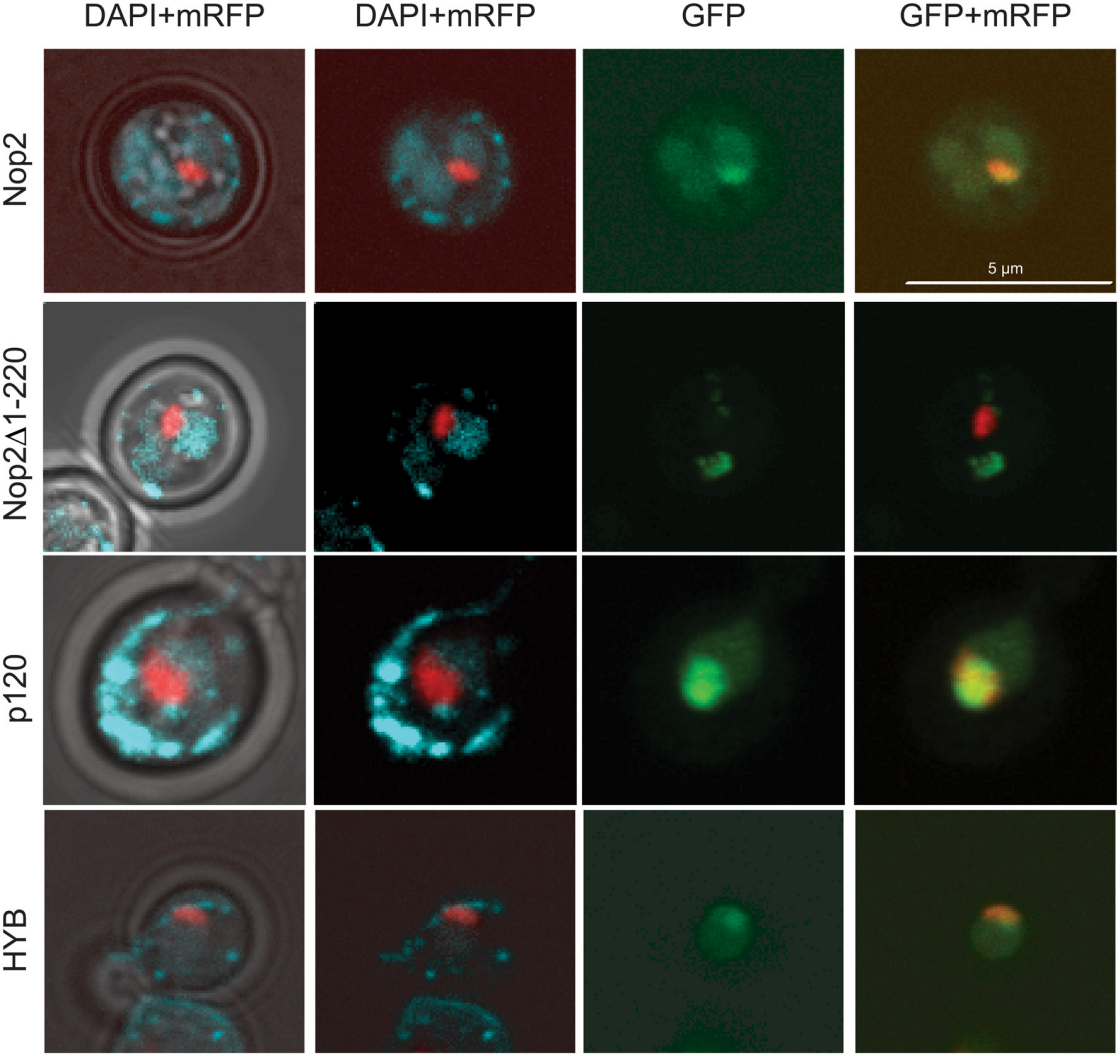
Subcellular localization of WT Nop2, p120, Nop2Δ1–220 and chimeric HYB protein. GFP-tagged variants were expressed in the presence of mRFP-Nop56 [46] and the endogenous Nop2 in yeast. Cells were DAPI stained and imaged *in vivo*. Columns from left to right: (1) merge of brightfield, DAPI and mRFP-Nop56 channels to establish the borders of the cells, the nuclei and nucleoli, respectively; (2) DAPI and mRFP-Nop56 only; (3) GFP-tagged protein as indicated to the left of the rows; and (4) merge between GFP-tagged protein and mRFP-Nop56 signals to highlight the possible co-localization of the two signals. The scale bar is 5 μm. Quantification of nuclear and nucleolar signals is given in the Table A in S1 Text.

The NTD of Nop2 contains predicted subcellular sorting signals (S1B Fig) [44]. Yeast strain expressing NTD-truncated Nop2 was not viable (Nop2**Δ**NTD), indicating that localization of Nop2 is important for its function. To investigate the importance of this domain for Nop2 subcellular localization, we generated a new truncated mutant of Nop2. Alignment of p120 and Nop2 sequences revealed a small conserved region upstream of the MT domain ([221–383] of Nop2). Therefore, the first 220 amino acid residues were removed from Nop2 (Nop2**Δ**(1–220)). Strains expressing Nop2**Δ**(1–220) were viable, but exhibited a significant growth defect (Fig 1B, tetrad 5). To check the subcellular localization of these variants in *S*. *cerevisiae*, we expressed N-terminally GFP-tagged p120, Nop2 and Nop2**Δ**(1–220), as well as the HYB protein in a WT yeast strain. WT Nop2 protein was strongly enriched in the nucleus and concentrated in the nucleolus as shown by colocalization with the mRFP-tagged nucleolar marker Nop56 (Sik1) [46]. Results presented in Fig 2 show an almost perfect overlap of mRFP-Nop56 and GFP-Nop2 signals. Deletion of the [1–220] domain of Nop2 resulted in the delocalization of the protein into the cytoplasm. These data show that the NTD domain of Nop2 is required for its nucleolar localization. Full-length human p120 when expressed in *S*. *cerevisiae* strongly accumulated in the nucleolus.

In contrast, the HYB protein, which is composed of N-terminal Nop2 and C-terminal p120 domains, efficiently co-localized with mRFP-Nop56 within the nucleolus, demonstrating that all necessary cell sorting signals are indeed located in the Nop2 N-terminal domain.

### Nop2 NTD is sufficient for rRNA binding

Yeast Nop2 was reported to be involved in the processing of the 35S rRNA precursor [43, 52] and its absence was shown to affect C1/C2 cleavages and thereby to decrease the level of mature 25S rRNA [43].

**Fig 3:**
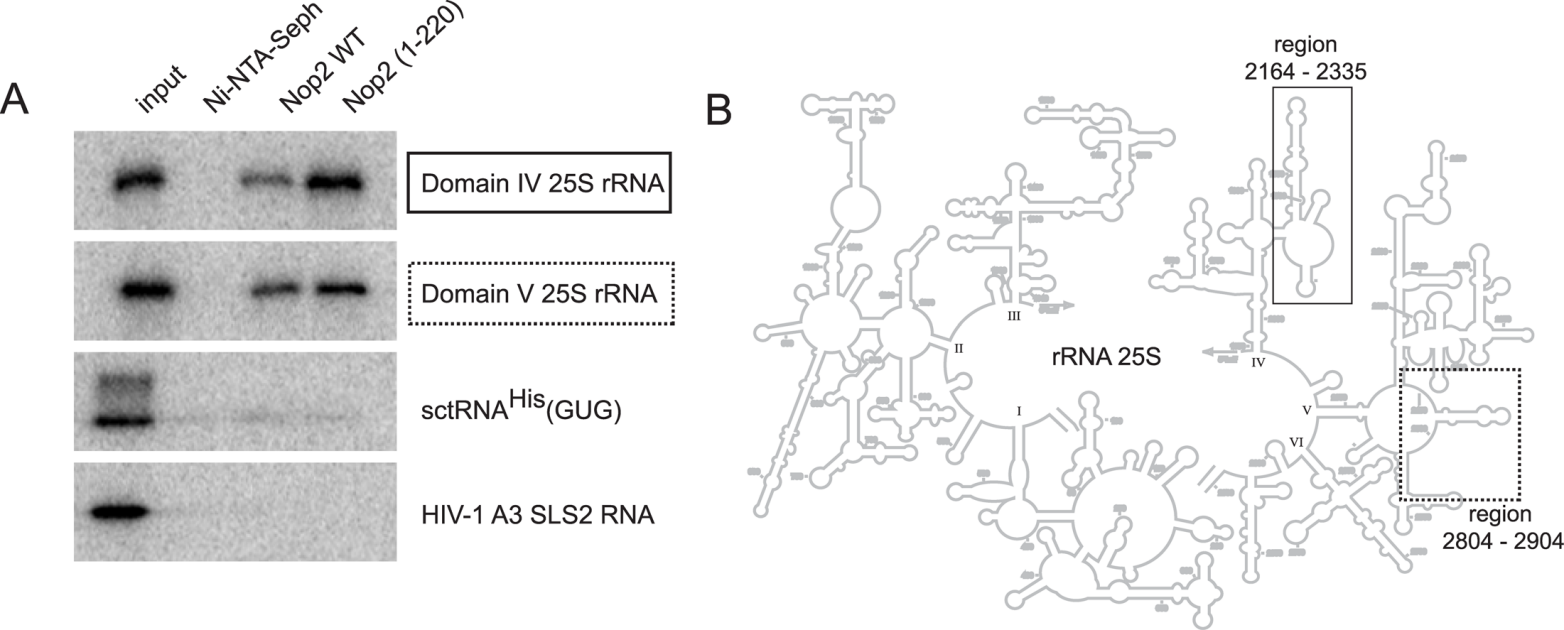
Specific rRNA binding by recombinant full-length Nop2 and its N-terminal fragment. (A) His_6_-tagged recombinant protein was immobilized on Ni-NTA Sepharose beads and incubated with radiolabeled transcripts of Domains IV and V of *S*. *cerevisiae* 25S rRNA (fragments spanning nucleotides 2164−2335 and 2804−2904, respectively), RNAs derived from *S*. *cerevisiae* tRNA^His^(GUG), HIV-1 derived RNA covering stem-loop structure 2 (SLS2) of the A3 splice site [47] and also transcripts corresponding to yeast ITS1 and ITS2 rRNA sequences. After extensive washing to remove unbound RNA, the retained fraction was directly loaded onto SDS-PAGE and analyzed by autoradiography. 5% of the input was loaded in the first lane on the left. The second lane shows the RNA bound without immobilized protein. (B) Schematic representation of 25S yeast rRNA: the well-defined structural domains are numbered from I to VI, and the regions corresponding to tested *in vitro* transcripts are boxed (solid box–Domain IV, dashed box–Domain V).

The NTD from human p120 contains an arginine-rich domain that tightly binds to ribosomal RNA [35]. It was previously predicted that NTD of Nop2 contains an RNA binding domain [53], however this prediction was not verified experimentally. Thus, we addressed the ability of Nop2 and of its NTD [1–220] domain to interact with different RNA molecules. Interaction studies were performed using retention assay of radiolabeled RNAs on His_6_-tagged-Nop2 and His_6_-tagged-Nop2 [1–220] immobilized on Ni-NTA Sepharose beads. As two m^5^C residues were recently identified in domain IV and V of rRNA 25S [7], both domains (spanning the regions 2164−2335 and 2804−2904, respectively) were chosen as RNA substrates produced by *in vitro* transcription. The results presented in Fig 3 show that the recombinant Nop2 protein binds to both 25S rRNA fragments, but not to other types of RNA, such as yeast tRNA^His^ or HIV-1 derived RNAs. Also, no significant binding was observed with synthetic RNA transcripts derived from ITS1 and ITS2 sequences either (data not shown). Identical results were obtained for the Nop2 [1–220] domain, indicating that this positively charged domain is sufficient to mediate RNA binding. Taken together, these results show that the NTD domain of Nop2 is involved in specific RNA binding.

### Human p120 restores the formation of m^5^C in domain V of yeast LSU rRNA

It was recently shown that yeast 25S rRNA contains two m^5^C residues at positions 2278 and 2870 and that the enzymes catalyzing these modifications are Rcm1 and Nop2, respectively [7, 8]. To investigate whether human p120 is a *bona fide* ribosomal RNA:MTase, we have analyzed for the presence of m^5^C in haploid strain *nop2Δ* that expressed p120 using mass spectrometry and RNA bisulfite sequencing.

**Fig 4:**
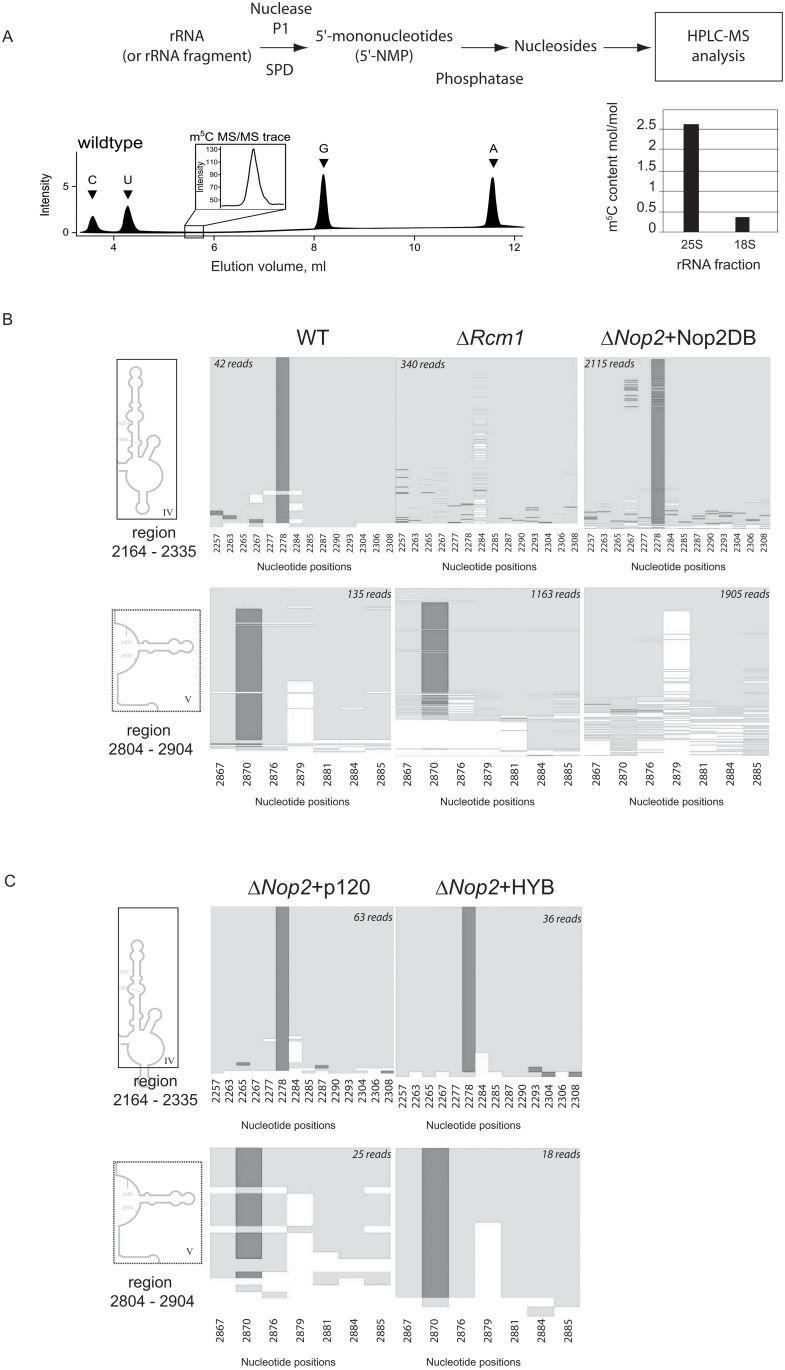
(A) Analysis of m^5^C presence in full length yeast rRNA. Schematic representation of the procedure is given on the top and typical separation profile for nucleosides at the bottom. Quantification of m^5^C residues in full-length 25S and 18S rRNAs is presented by the histogram. (B) Results of bisulfite sequencing of Domains IV and V of 25S rRNA extracted from different deleted and complemented yeast strains. Dark gray shade indicate bisulfite resistant non-deaminated m^5^C residue, light shade represents deamination events C → U. Numbering of all C residues present in the domain is given at the bottom of each panel. Number of reads mapped to a given region is indicated on the top. (C) Bisulfite sequencing of 25S rRNA extracted from yeast strains expressing human p120 and HYB proteins. The legend is the same as for panel B.

First, we analyzed the global m^5^C content in 25S rRNA and 18S rRNA from WT yeast *S*. *cerevisiae* using HPLC-MS/MS analysis. Precise quantification of m^5^C signals confirmed the presence of at least two m^5^C modification sites in 25S rRNA, while 18S rRNA contained only substoichiometric amounts (~0,25 mole/mole) of m^5^C (Fig 4A), possibly due to slight contamination by 25S rRNA degradation products. Next, we mapped the m^5^C residues in 25S rRNA by focusing on domains IV and V (regions encompassing residues 2235–2336 and 2844–2906, respectively). The results of RNA bisulfite sequencing are given in Fig 4B. Two bisulfite-resistant signals resulting from the presence of m^5^C residues were found at positions C2278 and C2870. Taken together, our results from HPLC-MS/MS analysis and RNA bisulfite sequencing unambiguously map two m^5^C residues at positions 2278 and 2870 of 25S rRNA and validate our experimental approach.

Next, we confirmed that Rcm1 is essential for formation of m^5^C2278 as this modification disappeared upon deletion of the recently characterized yeast ORF RCM1, while m^5^C2870 remained intact in this strain. As the deletion of NOP2 gene is lethal, we complemented a nop2Δ haploid strain with a plasmid expressing a Nop2 protein with two mutated cysteines that are expected to be involved in catalysis, namely Cys424 and Cys478 (Nop2DB, S2 Fig). One of the catalytic cysteines in the m^5^C-MTase active site is required for initial attack to target cytosine, while the second serves for recycling function during the product release (see [9] for review and S3 Fig). The mutation of the recycling cysteine is frequently deleterious for growth [53], while the consequences of replacement of the catalytic residue seem to depend on the strain background [7, 53]. Here, both catalytic and recycling cysteines were mutated to abolish m^5^C-MTase activity.

This double mutation in Nop2 caused no phenotypic effects, thus mapping of m^5^C residues in extracted 25S rRNA was done by RNA bisulfite sequencing. The results showed the absence of m^5^C2870, which confirmed that Nop2 is the enzyme responsible for the catalysis of this modification. Importantly, these results further indicate that the m^5^C modification at position 2870 is not required for rRNA maturation or cell viability [53].

Last, we performed an rRNA methylation analysis in the haploid strain *nop2Δ*, which expressed human p120 and the HYB protein bearing the MTD domain of p120. The expression of full-length p120 or the HYB proteins in nop2Δ strain completely restored the formation of m^5^C2870 in domain V of 25S rRNA (Fig 4C). These data clearly show that human p120 displays rRNA: m^5^C-MTase activity, which efficiently modifies 25S rRNA at position 2870 when expressed in *S*. *cerevisiae*. This data provides the first direct evidence for an RNA:m^5^C-MTase activity of human proliferation-associated antigen p120. Furthermore, this result also confirmed that the slow growth phenotype observed in the nop2Δ strain expressing human p120 did not result from a lack of m^5^C2870 modification in 25S rRNA. This indicates that (cytosine-5) methylation is not important for the function of 25S rRNA under steady-state conditions and that Nop2 function affects cell growth independently of its RNA methyltransferase function.

## Discussion

In this study, we provide the first evidence that human proliferation associated antigen p120 has an rRNA:m^5^C-MTase activity. Indeed, expression of full-length p120 and HYB protein (bearing p120 MTase domain) completely restored the formation of m^5^C2870 in domain V of 25S rRNA. The NTD of Nop2, which is essential for nucleolar localization and sufficient for RNA binding, is crucially important for ribosome synthesis, but not necessary for efficient rRNA methylation.

In contrast to results reported by Sharma *et al*. [7], we observed that the loss of m^5^C2870 in helix 89 of 25S rRNA had no effect on ribosome synthesis as shown by using the Nop2DB mutant in complementation assays. We conclude that the activity of the Nop2 RNA:m^5^C-MTase is not required for the function of Nop2 in pre-rRNA processing. These results also confirm that Nop2 belongs to the large family of pre-ribosomal protein and possesses two related functions: an rRNA modification activity and an essential role in pre-rRNA cleavage(s). These results corroborate the concept of quality control of ribosome synthesis by several modification enzymes. This notion was previously developed for Dim1p [54], then for Emg1 [55, 56] as well as for Bud23 [38, 39]. These rRNA-MTases bind to pre-rRNA in the nucleolus at an early stage in ribosome synthesis and allow coordinated cleavages in pre-rRNA while present in the nucleolus. Similarly, when Nop2p is bound to pre-rRNA, the processing machinery may identify this interaction allowing cleavage at sites C1/C2 to proceed; otherwise, processing is blocked. The strong growth phenotype that we have observed for p120 expressing yeast is probably related to the inability of the yeast pre-RNA processing machinery to use p120 instead of Nop2, possibly causing a delay in the 25S rRNA synthesis and thereby the observed slow growth phenotype.

The identity elements required for efficient pre-rRNA processing are likely to be localized in the NTD of Nop2, as suggested by near-complete complementation using HYB protein. This domain could be involved in the interaction with other ribosomal factors that are essential for fine-tuning of pre-rRNA processing. The majority of identified Nop2 protein partners (see http://genemania.org/) belong to the 66S assembly particle (e.g. Nip7, Erb1, Rrp1, Nop15) [57–59]. It is therefore likely that Nop2 may also play a role as an adapter protein (via its NTD domain containing coiled coil region 132–174), which is required for the recruitment and correct positioning of additional ribosomal factors on pre-RNA maturation intermediate. Studies on the mechanism of ribosomal assembly provide support for this hypothesis [60]. Nop2 tightly interacts with Nip7 and this interaction is required for assembly of other B-factors with nascent ribosomes. There are several other examples of RNA-MTases working as heterodimers or in more sophisticated complexes. For instance, this is the case for Bud23-Trm112 [39], Trm8-Trm82 [61] or Nsun4-MTERF4 [62, 63].

Thus far, among members of the NSUN family in human, only the enzymatic activities of NSUN2 [23, 64] and NSUN4 [24] had been partially characterized. Our study reports now the RNA: methyltransferase activity of p120 (NSUN1) on heterologous rRNA. A cytosine at position 4447 in human 28S rRNA is methylated [10, 49], which we predict to be the natural target of p120. Future biochemical experiments are required to confirm this and to find other putative substrates.

## Supporting Information

S1 Fig Comparative analysis of Nop2 and p120 amino acid sequences. (A) Conservation of the putative catalytic cysteine residues in Motif IV (PCS-sequence) and Motif VI (TCS-sequence) in MTD of Nop2 and p120. Catalytic motifs are boxed; identical amino acid residues are shaded. (B) Alignment of Nop2 and p120 NTDs. Experimentally confirmed nuclear and nucleolar localization signals (NLS[40–57] in bold characters and NoLS[99–110] is boxed [34]) are shown for p120. NLS which was suggested to drive Nop2 to the nucleus is shown in bold (_50_KKKSK_54_), while NoLS predicted by PredictProtein (http://www.predictprotein.org) is boxed.
(EPS)

S2 Fig Growth of individual spores expressing WT Nop2 and Nop2 DB mutant. (EPS)

S3 Fig Catalytic mechanism of RNA:m^5^C-MTases. (EPS)

S1 Text Legends for Supplementary figures and Table A. (DOC)
