# Supplementary material for: Eukaryotic rRNA Modification by Yeast 5-Methylcytosine-Methyltransferases and Human Proliferation-Associated Antigen p120
Source: PLoS One. 2015 Jul 21;10(7):e0133321. doi: 10.1371/journal.pone.0133321 (PMC4510066; doi:10.1371/journal.pone.0133321)
Supplement: S1 Text — (DOC) [file pone.0133321.s004.doc]

## Eukaryotic rRNA modification by yeast 5-methylcytosine-methyltransferases and human proliferation-associated antigen p120

Gabrielle Bourgeois^1^, Michel Ney^1^, Imre Gaspar^2^, Christelle Aigueperse^1^, Matthias Schaefer^3^, Stefanie Kellner^4^, Mark Helm^4^, Yuri Motorin^1*^

### **Supporting information**

**S1 Text** Legends for Supplementary figures and Table A

**Figure legends**

**S1 Fig. Comparative analysis of Nop2 and p120 amino acid sequences**

(A) Conservation of the putative catalytic cysteine residues in Motif IV (PCS-sequence) and Motif VI (TCS-sequence) in MTD of Nop2 and p120. Catalytic motifs are boxed; identical amino acid residues are shaded.

(B) Alignment of Nop2 and p120 NTDs. Experimentally confirmed nuclear and nucleolar localization signals (NLS[40-57] in bold characters and NoLS[99-110] is boxed (34)) are shown for p120. NLS which was suggested to drive Nop2 to the nucleus is shown in bold (_50_KKKSK_54_), while NoLS predicted by PredictProtein ([http://www.predictprotein.org](http://www.predictprotein.org/)) is boxed.

**S2 Fig.:** Growth of individual spores expressing WT Nop2 and Nop2 DB mutant.

**S3 Fig.:** Catalytic mechanism of RNA:m^5^C-MTases

**Table A** Relative nucleolar excess of GFP-Nop2 wt, p120 and chimeric protein HYB

## Quantification of subcellular localization

Table A **Relative nucleolar excess of GFP-Nop2 wt, p120 and chimeric protein HYB**

| Construct | Number of GFP positive cells/  number of cells analyzed | Rel. nucleolar excess of GFP signal (%, mean±SD) |
| --- | --- | --- |
| wt | 49/53 | 97.2±46.4 |
| p120 | 41/68 | 88.8±56.7 |
| HYB | 72/72 | 110.0±68.2 |

**Evaluation of the relative enrichment of GFP signals within the nucleolus.**

The nucleolar and the nuclear GFP signal was determined in cells expressing GFP tagged Nop2 variants using ImageJ. Nucleolar contours were determined based on mRFP-Nop56 signal, using automatic thresholding on the appropriate channel of the image. We used the combined DAPI and mRFP-Nop56 channels to determine the nuclear contours for more feasible identification of the usually weakly stained nucleus and for the inclusion of the nucleolus within the nuclear contours. The specified contours were applied to the corresponding GFP-Nop2 variant channel pairwise, measuring the contained mean intensity value and the excapsulated area. In the same channel a cell free area was defined to measure the mean intensity value of the background. This value was subtracted from the measured nuclear and nuclear mean intensities to correct for background. If the corrected mean intensity within the nucleolus did not exceed 3 (arbitrarily chosen) the analysis of the given cell was abandoned. The corrected mean intensities multiplied with the corresponding area yielded the background corrected total nuclear and nucleolar intensities. The nuclear area and thus the nuclear intensities contained nucleolar information by definition. This contamination was removed simply by subtracting the total nucleolar intensity for the total nuclear intensity and this product was used to estimate the exclusively nuclear GFP concentration (1). As the nucleolus is a non separated part of the nucleus we assumed that part of the nucleolar GFP signal is aspecificly present in the organelle, proportional to its size. After correcting with this factor (2), we obtained the GFP signal that is present in excess within the nucleolus. Taking their ratio (3) yielded in the relative nucleolar excess of the given GFP-Nop2 variant on top of the expected, aspecificly present amount. The resulting datasets were compared to each other using the non-parametric Kruskal-Wallis test the samples are not normally distributed. As the KW test suggested non equality of the datasets (p<0.001, a=0.05) we performed pairwise Mann Whitney U tests comparing the other variants to the distribution of wt Nop2.

*I_Nu, corr_, I_No, corr_ –* measured, background corrected total nuclear and nucleolar intensities

*A*Nu, *A*No – measured nuclear and nucleolar areas

*c*Nu – concentration of GFP signal in nuclear regions, excluding the nucleolus

*c*No – excess concentration of GFP signal within the nucleolus

## References

34. Valdez BC, Perlaky L, Henning D, Saijo Y, Chan PK, Busch H. Identification of the nuclear and nucleolar localization signals of the protein p120. Interaction with translocation protein B23. J Biol Chem 1994;269(38):23776-83.
